# Metastasis-directed stereotactic radiotherapy in patients with breast cancer: results of an international multicenter cohort study

**DOI:** 10.1007/s10585-024-10326-x

**Published:** 2024-12-21

**Authors:** Alexander Fabian, Daniel Buergy, Fabian Weykamp, Juliane Hörner-Rieber, Denise Bernhardt, Judit Boda-Heggemann, Montserrat Pazos, Nora Mehrhof, David Kaul, Alicia S. Bicu, Eugenia Vlaskou Badra, Susanne Rogers, Stefan Janssen, Hossein Hemmatazad, Katharina Hintelmann, Eleni Gkika, Tim Lange, Konstantinos Ferentinos, Heiko Karle, Thomas Brunner, Andrea Wittig, Marciana Nona-Duma, Oliver Blanck, David Krug

**Affiliations:** 1 Department of Radiation Oncology, University Hospital Schleswig-Holstein Campus Kiel, Arnold-Heller-Str.3, 24105 Kiel, Germany; 2 Department of Radiation Oncology, University Medical Center Mannheim, University of Heidelberg, Mannheim, Germany; 3 Department of Radiation Oncology, Heidelberg University Hospital, Heidelberg, Germany; 4 Clinical Cooperation Unit Radiation Oncology, German Cancer Research Center (DKFZ), Heidelberg, Germany; 5 Heidelberg Institute of Radiation Oncology (HIRO), 69120 Heidelberg, Germany; 6 National Center for Tumor Diseases (NCT), 69120 Heidelberg, Germany; 7 Department of Radiation Oncology, University Hospital Düsseldorf, Düsseldorf, Germany; 8 Department of Radiation Oncology, Technical University of Munich, Munich, Germany; 9 Department of Radiation Oncology, University Hospital, LMU Munich, Munich, Germany; 10 Department of Radiation Oncology, Charité—Universitätsmedizin Berlin, Berlin, Germany; 11 Department of Radiation Oncology, Health and Medical University Potsdam, Potsdam, Germany; 12 Department of Radiation Oncology, University Hospital & University of Zurich, Zurich, Switzerland; 13 Radiation Oncology Center KSA-KSB, Kantonsspital Aarau, Aarau, Switzerland; 14 Department of Radiation Oncology, University of Lubeck, Lubeck, Germany; 15 Medical Practice for Radiotherapy and Radiation Oncology, Hannover, Germany; 16 Department of Radiation Oncology, Inselspital, Bern University Hospital, University of Bern, Bern, Switzerland; 17 Department of Radiotherapy and Radiation Oncology, University Medical Center Hamburg-Eppendorf, Hamburg, Germany; 18 Department of Radiation Oncology, University Medical Center Freiburg, Freiburg, Germany; 19 Department of Radiation Oncology, University Hospital Bonn, University of Bonn, Bonn, Germany; 20 Clinic for Radiotherapy, Hannover Medical School, Hannover, Germany; 21 Department of Radiation Oncology, German Oncology Center, European University of Cyprus, Limassol, Cyprus; 22 Department of Radiation Oncology and Radiotherapy, University Medical Center, Mainz, Germany; 23 Department of Radiation Oncology, University Hospital Graz, Graz, Austria; 24 Department of Radiation Oncology, University Hospital Würzburg, Würzburg, Germany; 25 Department of Radiation Oncology, HELIOS Hospital Schwerin, Schwerin, Germany; 26 Department for Human Medicine, MSH Medical School Hamburg, Hamburg, Germany

**Keywords:** Breast cancer, Metastasis, Oligometastasic disease, Stereotactic radiotherapy, Brain metastases

## Abstract

Metastasis-directed therapy (MDT) for oligometastatic breast cancer (≤ 5 metastases) has shown little effect in specific scenarios of randomized trials. Therefore, we aimed to assess outcomes after metastasis-directed stereotactic radiotherapy (SRT) in various clinical scenarios. We conducted an international retrospective cohort study in thirteen centers including breast cancer patients receiving SRT to any metastatic site. Outcomes included local recurrence (LR), progression-free survival (PFS), and overall survival (OS). Cumulative incidence analysis was used for LR, Kaplan–Meier estimates for PFS and OS. Covariables included patient, disease, and SRT characteristics. We performed univariable and multivariable analyses (MVA). Among 444 patients, 751 metastases were treated with SRT. Of these, 73% were intracranial and 27% extracranial lesions. Oligometastatic disease (OMD) was present in 66% of the patients. LR after two years occurred significantly more often in intracranial (25%) versus extracranial lesions (7%). In MVA of patients with OMD treated for intracranial sites, higher performance status was significantly associated with longer PFS. Further, higher performance status, biologic subtype (HR-pos./HER2-pos.), and MDT to all sites were significantly associated with longer OS. In MVA of oligometastatic patients treated for extracranial sites, biologic subtype (HR-neg./HER2-pos.) and synchronous metastasis were associated with significantly longer PFS, whereas higher grading was associated with significantly shorter PFS. Moreover, biologic subtype (HR-neg./HER2-neg.) was associated with significantly shorter OS. In conclusion, the role of MDT for breast cancer may vary per clinical scenario. Patients with OMD treated for intracranial lesions who had MDT to all sites showed superior OS. Our results should be validated prospectively.

**Supplementary Information:**

The online version contains supplementary material available at 10.1007/s10585-024-10326-x.

## Introduction

Stereotactic radiotherapy (SRT) has been used to treat patients with brain metastases for several decades [1–3]. Randomized-controlled trials and meta-analyses have established that treatment with SRT alone achieves equivalent overall survival (OS) compared to SRT with whole-brain radiotherapy for patients with limited brain metastases [4]. Due to the lack of OS improvement and neurocognitive impairment with whole-brain radiotherapy, SRT is increasingly used for patients with up to 10 or even > 10 brain metastases [5]. Since the concept of oligometastatic disease was introduced by Hellman and Weichselbaum in 1995, there has been an increasing interest in the use of metastases-directed therapy (MDT) to improve the prognosis of patients in this disease state [6]. The randomized controlled SABR-COMET phase II-trial provided a proof-of-concept that delivering SRT to all disease sites in patients with up to 5 metastases improves progression-free survival and OS [7]. However, this trial was criticized for including all histologies and primaries. In several pooled analyses, breast cancer as primary emerged as a positive prognostic factor [8]. Further, several prospective phase II-trials and a meta-analysis have reported excellent local control and progression-free survival (PFS) rates with SRT for patients with oligometastatic breast cancer [9–13]. Yet the randomized phase II trial NRG-BR002 and EXTEND-trials failed to demonstrate an improvement in progression-free survival with the addition of SRT to standard systemic therapy in patients with breast cancer and up to 4 metastatic lesions [14, 15]. Similarly, addition of SRT to standard therapy failed to improve PFS in patients with oligoprogressive breast cancer in the randomized controlled phase II CURB-trial [16]. Most of these trials, however, focused on specific clinical scenarios. Patients with intracranial metastases, for example, were often excluded or underrepresented. Thus, further research is necessary to understand the effects of SRT in patients with metastatic breast cancer in various clinical scenarios to improve treatment strategies and patient selection for these approaches.

## Materials and methods

### Study design

We conducted an international retrospective multicenter cohort study within the German Society for Radiation Oncology (DEGRO) working group for radiosurgery and stereotactic radiotherapy. Approval from local ethics committees was acquired for each participating center after approval for the leading study center (Kiel D582/20). Thirteen academic centers and one non-academic center from Germany, Switzerland, and Cyprus contributed data from all potentially eligible patients treated from 02/2002 until 05/2021. The primary objective was to investigate the efficacy of metastasis directed SRT in breast cancer patients in terms of oncological outcomes (local recurrence, PFS, OS) in various clinical scenarios.

Eligible patients had histologically confirmed breast cancer and had at least one treatment course with SRT to at least one metastatic site. In case of multiple courses of SRT, only the first course was analyzed for this report. The minimal dose of SRT was defined as biologically effective dose (α/β = 10 Gy) of at least 45 Gy delivered over a maximum of 12 treatment sessions [2]. The STROBE guideline was respected for reporting the study as applicable [17].

### Variables and endpoints

Data was collected based on medical records using a predefined data extraction form as in previous comparable studies [18–21]. It covered variables on characteristics of patients (e.g. performance status per Karnofsky performance status), their breast cancer disease (e.g. biologic subtype), systemic therapy (e.g. active systemic therapy at SRT), and SRT (e.g. dose, fractionation, imaging). Patients were considered to have oligometastatic disease in case of no more than five metastases. Synchronous metastatic disease was defined as detection of metastases within six months after initial breast cancer diagnosis.

Endpoints included rate of local recurrence of metastatic sites treated with SRT, PFS, OS, and toxicity per Common Terminology Criteria for Adverse Events (CTCAE) v5.0. Toxicity was defined as acute toxicity (≤ 90 days from SRT) or late toxicity (> 90 days from SRT).

### Statistical analysis

Descriptive statistics were used to display the study cohort. All analyses were exploratory. Time-dependent endpoint analyses were investigated from the last fraction of SRT until the occurrence of a respective event. Local recurrence of metastases treated in a first course of SRT was estimated using a cumulative incidence function in which death was considered a competing event. Patients without available follow-up data on local recurrence were excluded from the analysis unless they died less than eight weeks after SRT in which case absence of local recurrence was assumed. This approach was chosen because patients typically receive a first follow-up imaging eight weeks after SRT, and short-term local control rates are excellent [22]. Local recurrence was assessed per metastasis. Differences in local recurrence between groups were assessed using Gray’s test. A further in-depth analysis on factors potentially associated with local recurrence (e.g. SRT dose) is not the focus of the presented manuscript and will be reported separately. PFS included local recurrence, distant progression, or death as potential events. PFS and OS were analyzed using the Kaplan–Meier method. The log-rank test was used to assess differences in PFS and OS between groups. Patients lacking data on local or distant recurrences were excluded from the PFS analysis. To control for potential confounders in univariable results, we conducted multivariable Cox regression models for PFS and OS. The assumption of proportional hazards was violated in models containing the whole cohort as assessed by interaction terms. Therefore, we stratified “SRT to intracranial lesions in oligometastatic patients” and “SRT to extracranial lesions in oligometastatic patients” as separate subgroups of interest meeting the assumption of proportional hazards in each model [23]. Patients in each of these groups may have had additional intra- or extracranial metastases and vice versa. No patient was treated with SRT to intra- as well as extracranial lesions in a first course of SRT. MDT to all known metastases also included local treatment modalities other than SRT. Model covariables were entered in a single step and chosen based on their known or assumed clinical influence on PFS and OS. A two-sided p-value < 0.05 was considered statistically significant. Patients with missing data were excluded from respective analyses. All analyses were performed with JASP v0.17.2.1 (JASP Team [2022], Amsterdam, the Netherlands) or R (version 4.3.3; the R Foundation for Statistical Computing, Vienna, Austria).

## Results

### Characteristics of the study cohort

A total of 564 patients with 1250 metastases treated with SRT were entered into the database (Supplementary Fig. 1). Accounting for eligibility and follow-up data, 444 patients treated in a first course of SRT for 751 metastases were available for analysis. The median follow-up period for OS and PFS analyses were 15.6 and 7.3 months, respectively.

Table 1 displays patient characteristics at initial diagnosis of breast cancer and at the time of SRT. In brief, the median age was 51 years (interquartile range (IQR), 42–59) at initial diagnosis and 58 years (IQR, 49–67) at SRT. Oligometastatic disease at SRT was present in 66% (294/444) of the patients. Supplementary Table 1 shows characteristics of SRT treatments. SRT was performed for intracranial lesions in 73% (547/751) and extracranial lesions in 27% (204/751) of the treated metastases, respectively. The most common intracranial lesions were intact brain metastases at 64% (482/751), whereas bone metastases were the most commonly treated extracranial lesions at 13% (96/751).

**Table 1:** Characteristics of patients (n = 444) at initial diagnosis and at a first course of SRT for metastasis of breast cancer

Initial diagnosis	
Age	
Years	Median: 51 (IQR 42–59)
Missing	< 1% (1/444)
T-stage	
T1-2	64% (283/444)
T3-4	18% (80/444)
Missing	18% (81/444)
N-stage	
N0	31% (137/444)
N +	52% (231/444)
Missing	17% (76/444)
M-stage	
M0	72% (321/444)
M1	21% (94/444)
Missing	7% (29/444)
Biological Subtype	
HR + /HER2-	37% (168/444)
HR + HER2 +	19% (84/444)
HR-/HER2 +	14% (61/444)
HR-/HER2-	16% (69/444)
Missing	14% (62/444)
Grading	
G1	3% (15/444)
G2	37% (166/444)
G3	41% (183/444)
Missing	18% (80/444)
Initial treatment in curative intent	
Yes	80% (356/444)
No	17% (77/444)
Missing	3% (11/444)
Surgery—Breast	
Lumpectomy	50% (220/444)
Mastectomy	42% (185/444)
None	7% (33/444)
Missing	1% (6/444)
Surgery—ALND	
Yes	57% (255/444)
No	40% (177/444)
Missing	3% (12/444)
Breast radiotherapy—adjuvant	
Yes	68% (303/444)
No	27% (119/444)
Missing	5% (22/444)
Chemotherapy—(neo-)adjuvant	
Yes	73% (322/444)
No	25% (112/444)
Missing	2% (10/444)
Endocrine therapy—adjuvant	
Yes	52% (231/444)
No	41% (184/444)
Missing	7% (29/444)
First course of SRT	
Age	
Years	Median: 58 (IQR 49–67)
Missing	1% (2/444)
Performance status	
Karnofsky scale	Median: 80 (IQR 80–90)
Missing	5% (21/444)
PET-CT staging	
Yes	11% (48/444)
No	82% (366/444)
Missing	7% (30/444)
Controlled primary	
Yes	90% (401/444)
No	8% (35/444)
Missing	2% (8/444)
Number of metastases treated	
Overall	Mean: 1.7 (SD 1.3)
Intracranial	Mean: 1.9 (SD 1.5)
Extracranial	Mean: 1.3 (SD 0.7)
Missing	0% (0/444)
Number of metastases present	
1	28% (124/444)
2	17% (76/444)
3	9% (41/444)
4	7% (32/444)
5	5% (21/444)
> 5	29% (130/444)
Missing	5% (20/444)
Oligometastasic disease (1–5 Mets)	
Yes, synchronous^a^	7% (32/444)
Yes, metachronous	59% (262/444)
No	29% (130/444)
Missing	5% (20/444)
Metastasis-directed therapy to all known metastases	
Yes	37% (163/444)
No	62% (276/444)
Missing	1% (5/444)
Systemic therapy ± 4 weeks from RT	
Yes	64% (286/444)
No	32% (142/444)
Missing	4% (16/444)
Lines of prior palliative systemic therapy	Median: 1 (IQR 1–3)
Missing	44% (196/444)
Subsequent change in systemic therapy	
Yes	27% (121/444)
No	29% (128/444)
Missing	44% (249/444)

Percentages may not add up to 100 due to rounding error

*ALND* axillary lymph node dissection; *HR* hormone receptor; *IQR* interquartile range; *RT* radiotherapy; *SRT* stereotactic radiotherapy

^a^ ≤ 6 months from diagnosis

### Local recurrence

**Fig. 1:**
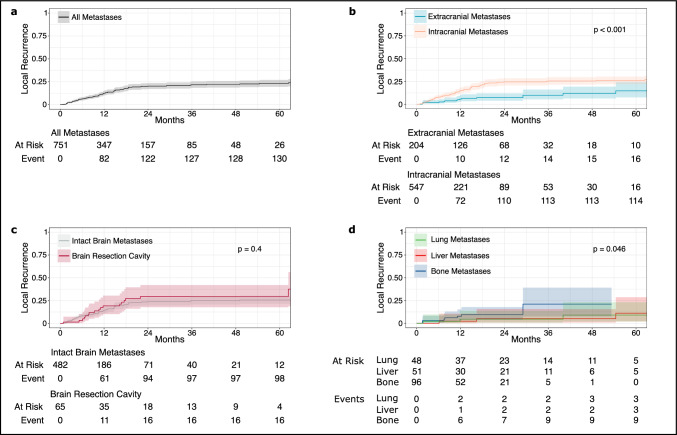
Local recurrence. Local recurrence rates after stereotactic radiotherapy are shown per metastasis for all analyzed metastases (**a**), extra- vs. intracranial metastases (**b**), intact brain metastases vs. brain resection cavities (**c**), and extracranial lung vs. liver vs. bone metastases (**d**). Analyses are based on cumulative incidence functions in which death was considered as a competing event

Cumulative incidence of local recurrence across metastatic sites was 13% (95% CI (confidence interval): 10–15%) and 20% (95% CI 17–24%) after 12 and 24 months, respectively (Fig. 1a). Cumulative incidence of local recurrence for extracranial lesions was 5.8% (95% CI 3–10%) and 7.3% (95% CI 4–12%) after 12 and 24 months, respectively. Compared to extracranial lesions, intracranial lesions had significantly higher rates of recurrence at 15% (95% CI 12–18%) and 25% (95% CI 21–29%) after 12 and 24 months, respectively (p < 0.001; Fig. 1b). Cumulative incidence of local recurrence after SRT for intact brain metastases and brain resection cavities was comparable (p = 0.4; Fig. 1c). Intact brain metastases recurred at 14% (95% CI 11–18%) and 24% (95% CI 20–28%) after 12 and 24 months, respectively. Local recurrence at resection cavities occurred at 19% (95% CI 10–30%) and 29% (95% CI 18–42%) after 12 and 24 months, respectively. Cumulative incidences of local recurrence rates of extracranial bone, liver and lung metastases are shown in Fig. 1d. After 12 months, local recurrence rates were 7.8% (95% CI 3–15%), 2.3% (95% CI 0.2–11%) and 4.5% (95% CI 0.8–14%) for bone, liver and lung metastases, respectively. After 24 months, local recurrence rates were 9.6% (95% CI 4–18%), 5.2% (95% CI 0.9–16%) and 4.5% (95% CI 0.8–14%) for bone, liver and lung metastases, respectively. Lymph node metastases and other lesions are not shown due to small numbers. Compared to bone metastases, visceral metastases (lung and liver metastases combined) showed significantly lower recurrence rates at 3.4% (95% CI 0.9–8.8%) and 4.8% (95% CI 2–11%) after 12 and 24 months, respectively (p = 0.046).

### Progression-free survival

**Fig. 2:**
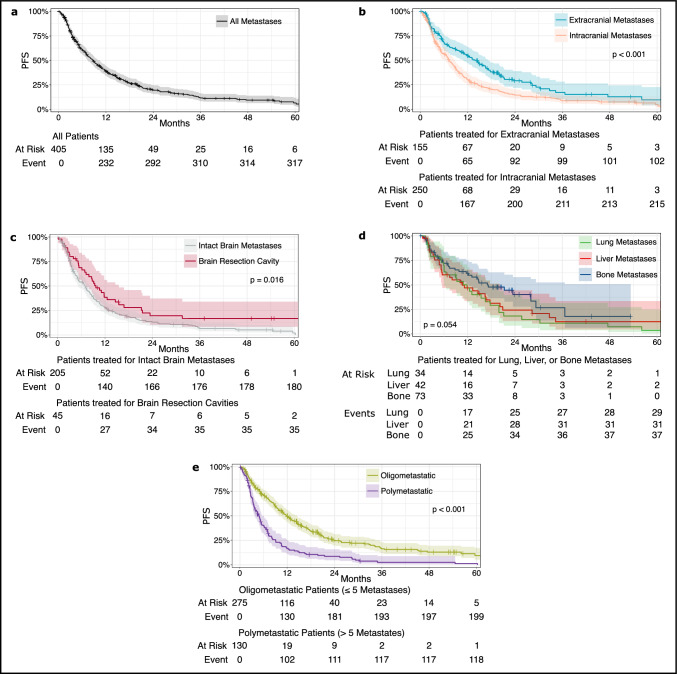
Progression-free survival. Progression-free survival (PFS) after stereotactic radiotherapy is shown for patients treated for any metastasis (**a**), extra- vs. intracranial metastases (**b**), intact brain metastases vs. brain resection cavities (**c**), extracranial lung vs. liver vs. bone metastases (**d**), and oligo- vs. polymetastatic patients (**e**). Abbreviation: PFS, progression-free survival

Median PFS in all patients with data on local and distant recurrence was 8.7 months (Fig. 2a; 95% CI 7–11 months). At 12 and 24 months, 39.0% and 19.6% of patients were alive without recurrence, respectively.

As per univariable analyses, patients who received SRT to intracranial metastases had significantly shorter PFS compared to patients who had SRT to extracranial metastases (Fig. 2b; 7.3 (95% CI 6–9) vs. 13.8 (95% CI 11–17) months; < 0.001). In patients with SRT to intracranial lesions, PFS was worse in those who had SRT for intact brain metastases compared to SRT for brain resection cavities (Fig. 2c; 6.9 (95% CI 5–8) vs. 9.7 (95% CI 7–15) months; p = 0.016). In patients with SRT to extracranial metastases, PFS showed no significant difference between bone metastases (PFS 17.0 (95% CI 12-not reached) months) and visceral metastases lung and liver combined; 10.9 (95% Cl 8–17) months; p = 0.054). Median values for lung and liver were 12.0 (95% CI 6–20) and 10.7 (95% CI 6–20) months, respectively (Fig. 2d). Patients with oligometastatic disease had superior median PFS compared to patients with polymetastatic disease (Fig. 2E; 11.8 (95% CI 10–15) vs. 4.8 (95% CI 4–6) months; p < 0.001). Patients who received MDT to all metastatic sites had superior median PFS compared to patients with MDT to selected sites (14.6 (95% CI 11–18) vs. 6.6 (95% CI 5–8) months; p < 0.001).

As per multivariable analysis in the cohort of oligometastatic patients treated for intracranial lesions, higher Karnofsky Performance status was significantly associated with longer PFS (hazard ratio (HR) = 0.976, 95% CI 0.958—0.995; p = 0.015) (Table 2). In the cohort of oligometastatic patients treated for extracranial lesions, biologic subtype (HR-neg./HER2-pos.) (HR = 0.240; 95% CI 0.06—0.965; p = 0.044) and synchronous metastatic disease (HR = 0.292; 95% CI 0.118—0.721; p = 0.008) were significantly associated with longer PFS (Table 3). Higher grading (HR = 2.066; 95% CI 1.106—3.857; p = 0.023) was significantly associated with shorter PFS. MDT to all sites was not associated with PFS in any of both subgroups.

**Table 2:** Progression-free and overall survival after stereotactic radiotherapy for intracranial metastases of oligometastatic breast cancer as per multivariable Cox regression analysis

	Progression-free survival				Overall survival			
	HR	Lower 95% CI	Upper 95% CI	*p*	HR	Lower 95% CI	Upper 95% CI	*p*
Age at initial Diagnosis	1.006	0.985	1.028	0.587	1.033	1.006	1.060	**0.015**
Biologic subtype (HR-pos./HER2-neg.)^a^	Reference				Reference			
Biologic subtype (HR-pos./HER2-pos.)	0.685	0.368	1.277	0.234	0.434	0.198	0.951	**0.037**
Biologic subtype (HR-neg./HER2-pos.)	1.082	0.591	1.982	0.798	1.019	0.505	2.054	0.958
Biologic subtype (HR-neg./HER2-neg.)	1.460	0.804	2.649	0.213	1.114	0.579	2.142	0.747
Grading	0.835	0.551	1.267	0.397	1.243	0.772	2.003	0.371
Karnofsky Performance Status	0.976	0.958	.995	**0.015**	0.958	0.937	0.979	**< 0.001**
Intact Brain Metastasis (1 = yes)^b^	1.031	0.556	1.914	0.922	1.327	0.615	2.864	0.471
Synchronous met. disease (1 = yes)^c^	1.726	0.597	4.989	0.313	3.123	0.978	9.973	0.055
Number of Metastases	1.104	0.853	1.429	0.451	1.131	0.848	1.508	0.402
All Metastases ablated (1 = yes)^d^	0.684	0.386	1.211	0.192	0.513	0.267	0.986	**0.045**

Statistically significant p-values < 0.05 are displayed in bold font

*CI* confidence interval; *HR* hazard ratio

^a^The covariable „Biologic subtype “ was dummy coded and “HR-pos./HER2-neg” was set as reference

^b^Binary variable “Intact Brain Metastasis” vs. “Brain resection cavity”

^c^Refers to diagnosis of metastatic disease within 6 months of initial breast cancer diagnosis

^d^Including all known present intra- and/or extracranial metastases

**Table 3:** Progression-free and overall survival after stereotactic radiotherapy for extracranial metastases of oligometastatic breast cancer as per multivariable Cox regression analysis

	Progression-free survival				Overall survival			
	HR	Lower 95% CI	Upper 95% CI	*p*	HR	Lower 95% CI	Upper 95% CI	*p*
Age at initial Diagnosis	1.006	0.978	1.035	0.655	1.021	0.976	1.067	0.363
Biologic subtype (HR-pos./HER2-neg.)^a^	Reference				Reference			
Biologic subtype (HR-pos./HER2-pos.)	2.113	0.859	5.196	0.103	0.550	0.083	3.664	0.537
Biologic subtype (HR-neg./HER2-pos.)	0.240	0.060	0.965	**0.044**	0.160	0.015	1.731	0.131
Biologic subtype (HR-neg./HER2-neg.)	1.817	0.797	4.142	0.155	5.524	1.703	17.922	**0.004**
Grading	2.066	1.106	3.857	**0.023**	2.593	0.988	6.805	0.053
Karnofsky Performance Status	0.999	0.960	1.041	0.978	1.048	0.974	1.127	0.213
Bone Metastasis (1 = yes)^b^	0.672	0.332	1.362	0.270	0.398	0.110	1.445	0.162
Synchronous met. disease (1 = yes)^c^	0.292	0.118	0.721	**0.008**	0.360	0.079	1.636	0.186
Number of Metastases	1.212	0.884	1.661	0.232	0.645	0.370	1.125	0.122
All Metastases ablated (1 = yes)^d^	0.660	0.339	1.282	0.220	1.541	0.427	5.563	0.509

Statistically significant p-values < 0.05 are displayed in bold font

*CI* confidence interval; *HR* hazard ratio

^a^The covariable „Biologic subtype “ was dummy coded and “HR-pos./HER2-neg” was set as reference

^b^Binary variable “Bone Metastasis” vs. “Visceral Metastasis (Lung and Liver Metastases)”

^c^Refers to diagnosis of metastatic disease within 6 months of initial breast cancer diagnosis

^d^Including all known present intra- and/or extracranial metastases

### Overall survival

**Fig. 3:**
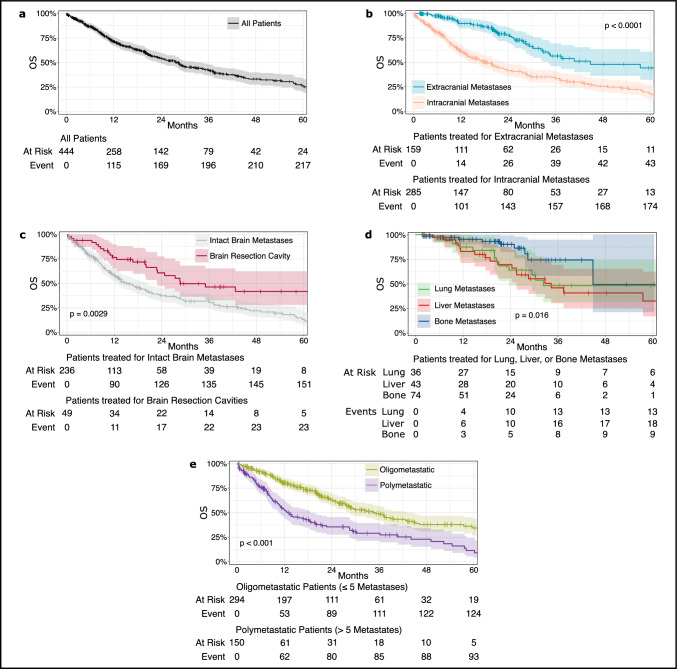
Overall survival. Overall survival (OS) after stereotactic radiotherapy is shown for patients treated for any metastasis (**a**), extra- vs. intracranial metastases (**b**), intact brain metastases vs. brain resection cavities (**c**), extracranial lung vs. liver vs. bone metastases (**d**), and oligo- vs. polymetastatic patients (**e**). Abbreviation: OS, overall survival

Median OS in the whole cohort of patients was 28.4 months (Fig. 3a; 95% CI 23.4–34.3). At 12 months, 71.4% of patients were alive. As per univariable analyses, patients who had received their initial SRT for intracranial metastases had significantly inferior OS compared to patients who were treated for extracranial metastases (Fig. 3b; median OS: 18.5 (95% CI: 14–23) vs. 44.8 (95% CI 35–85) months p < 0.0001). In patients with SRT to intracranial lesions, OS was significantly worse in those who had SRT for intact brain metastases compared to SRT for brain resection cavities (Fig. 3c; median OS: 15.1 (95% CI 13–20) vs. 28.7 (95% CI 23-not reached) months; p = 0.0029). In patients with extracranial lesions, bone metastases were associated with longer OS (median 44.8 (95% CI 45-not reached) months) compared to patients with visceral lesions (Fig. 3d; 32.7 (95% CI 29–73) months; p = 0.016). In those treated for visceral lesions, survival outcomes were similar between lung and liver metastases (32.3 (95% CI 24-not reached) and 34.3 (95% CI 26-not reached) months, respectively). Patients with oligometastatic disease had a significantly longer OS compared to patients with polymetastatic disease (Fig. 3E; 35.1 (95% CI 29–43) vs. 13.2 (95% CI 11–19) months; p < 0.001). Patients who received MDT to all metastatic sites had superior OS compared to patients who had MDT to selected sites (39.5 (95% CI 34–76) vs. 20.1 (95% CI 17–28) months; p < 0.001).

As per multivariable analysis in the cohort of oligometastatic patients treated for intracranial lesions, biologic subtype (HR-pos./HER2-pos.) (HR = 0.434; 95% CI 0.198–0.951; p = 0.037), higher Karnofsky Performance status (HR = 0.958, 95% CI 0.937–0.979; p < 0.001), and MDT to all known metastatic sites (HR = 0.513, 95% CI 0.267–0.986; p = 0.045) were significantly associated with longer OS (Table 2). Higher age at initial diagnosis (HR = 1.033, 95% CI 1.006–1.060; p = 0.015) was associated with shorter OS. In the cohort of oligometastatic patients treated for extracranial lesions, biologic subtype (HR-neg./HER2-neg.) (HR = 5.524; 95% CI 1.703—17.922; p = 0.004) was significantly associated with shorter OS (Table 3). MDT to all known metastatic sites was not associated with OS in the subgroup of treated extracranial metastases.

### Toxicity

Acute toxicity of CTCAE ≥ Grade 3 was present in 1.4% of the patients (6/444). Of these, one patient died from a suspected intracranial hemorrhage potentially associated with SRT. Autopsy was not performed. Late toxicity of CTCAE ≥ Grade 3 was present in 1.8% of patients (8/444). Among these, no treatment-related death occurred.

## Discussion

To our knowledge, we present data from the largest retrospective cohort of breast cancer patients treated with SRT to any metastatic site [8]. We report local recurrence rates, PFS, and OS in various clinical scenarios such as different intra- or extracranial treated lesions, oligo- or polymetastatic disease, and whether all known metastases were treated locally. This data aims to inform gaps in our current knowledge on the role of SRT in these different clinical scenarios.

### Progression analyses

Local recurrence rates for intracranial lesions were 15% at 1 year and 25% at 2 years after SRT, aligning well with outcomes reported in the literature [24, 25]. In the seminal study by Kocher and colleagues on the role of whole brain radiation versus observation after surgery or radiosurgery, for example, breast cancer was the second most common histology [25]. In this study, 31% of intracranial metastases treated with radiosurgery alone recurred after 2 years. Concerning extracranial lesions treated with SRT in our cohort, 1-year and 2-year local recurrence rates were 5.8% and 7.3%, respectively. These results are consistent, or slightly better than, previous results [8, 26]. A meta-analysis of extracranial breast cancer metastases treated with SRT reported local recurrence rates after 2 years of 10% and a recent retrospective cohort study reported local recurrences rates at 15% after 2 years [9, 27].

Randomized controlled trials conducted thus far did not report a benefit of SRT in terms of PFS or OS for breast cancer patients with oligometastatic disease. These trials often excluded or underrepresented patients treated with SRT for intracranial lesions and our data offers valuable insights in this context. Our univariable analyses suggested that longer PFS was present in patients treated for extracranial- versus intracranial metastases, SRT to brain resection cavities versus intact brain metastases, bone versus visceral metastases, and oligo- versus polymetastatic disease. For methodological reasons, multivariable analysis was only deemed feasible and informative in two separate cohorts. Oligometastatic patients treated for intracranial disease had longer PFS with better performance status. In contrast, oligometastatic patients treated for extracranial disease had longer PFS depending on biologic subtype, lower tumor grading and if synchronous metastatic disease was present. Notably, neither the variable SRT to brain resection cavities versus to intact brain metastases nor bone versus visceral metastases were significantly associated with PFS in the multivariable analysis suggesting potential confounders in the univariable analysis. Furthermore, although the hazard ratios of the multivariable analysis of PFS for patients who had MDT to all known metastases were in favor of the intervention, this effect was not statistically significant. This result is finally in line with previously mentioned data from randomized trials [14–16]. Treatment only to selected sites suggests that patients were either in a state of oligoprogression or symptomatic metastases were present. Oligoprogressive disease has been associated with an inferior prognosis when compared to oligometastatic disease [28]. However, recent data in patients with luminal-like tumors that developed oligoprogressive disease when treated with endocrine therapy and CDK4/6-inhibitors with SRT to oligprogressive lesions suggest that this approach may be reasonable to prolong time to the next line of systemic therapy [29].

### Overall survival analyses

Concerning OS, our univariable data suggested that longer OS was present in patients treated for extracranial- versus intracranial metastases, SRT to brain resection cavities versus intact brain metastases, bone versus visceral metastases, and oligo- versus polymetastatic disease. Our results on longer OS in patients with extracranial metastases, bone metastases, and oligometastatic disease seem plausible as these associations have been reported earlier [30]. The highly significant result of longer OS in patients treated with SRT to brain resection cavities compared to intact brain metastases is less clear [25]. In fact, this association was not present in our multivariable analysis of oligometastatic patients receiving SRT to intracranial lesions. This analysis showed that longer OS was associated with lower age at diagnosis, biologic subtype, better performance status, and MDT to all known metastases. In contrast, in oligometastatic diseases treated for extracranial disease only biologic subtype was significantly associated with OS in the multivariable analysis.

The result that MDT therapy to all known metastatic sites was associated with a longer OS in patients treated with SRT to intracranial lesions is thought provoking, especially as these patients were underrepresented in randomized trials thus far as mentioned above. Perhaps, these patients may benefit most from MDT as they tend to have worse survival outcomes and as traditionally systemic therapy was less active intracranially. This result should be interpreted with caution however, as many modern systemic agents show better intracranial antitumor activity [5].

### Limitations

To our knowledge, this analysis represents the largest cohort study of patients with metastatic breast cancer treated with SRT. Inclusion of patients with brain metastases allowed for a broad analysis of prognostic factors. Still limitations of this dataset include its retrospective design, which may result in incomplete data and follow-up. Due to the retrospective design, no standardized follow-up imaging was conducted. In analyzing primary vs. postoperative SRT for brain metastases, outcome differences may be influenced by selection bias. Diagnosis of local recurrence was not standardized. The cohort is heterogeneous in terms of patient characteristics and treatment details. We included regimens with a biologically equivalent dose (α/β = 10 Gy) as low as 45 Gy. While this dose may be considered sub-ablative, previous analysis of our working group suggest a shallow dose–response-relationship for SRT of breast cancer lung and liver metastases [31, 32]. Further analysis of dose–effect-relationship is planned in the future.

## Conclusions

In conclusion, patients treated with SRT for breast cancer metastases showed different outcomes in varying clinical scenarios. Despite limitations inherent to our study design, our data does generate hypotheses. Local recurrence appears to be more frequent in intracranial metastases as compared to extracranial metastases. Oligometastatic patients treated with SRT for intracranial lesions and MDT for all known metastases may experience superior OS compared to those with selected MDT, as shown in a multivariable model. Prospective studies are needed to validate these findings.

## Supplementary Information

Below is the link to the electronic supplementary material.

Supplementary file1 (PDF 111 KB)

## Data Availability

No datasets were generated or analysed during the current study.

## Abbreviations

DEGRO: German Society for Radiation Oncology
CTCAE: Common Terminology Criteria for Adverse Events
HR-neg: Hormone receptor negative
HR-pos: Hormone receptor positive
MDT: Metastasis-directed therapy
OMD: Oligometastatic disease
OS: Overall survival
PFS: Progression-free survival
SRT: Stereotactic radiotherapy
