# Supplementary material for: Metastasis-directed stereotactic radiotherapy in patients with breast cancer: results of an international multicenter cohort study
Source: Clin Exp Metastasis. 2024 Dec 21;42(1):6. doi: 10.1007/s10585-024-10326-x (PMC11663153; doi:10.1007/s10585-024-10326-x)
Supplement: Supplementary file 1 — Supplementary file1 (PDF 111 KB) [file 10585_2024_10326_MOESM1_ESM.pdf]

Metastasis-directed stereotactic radiotherapy in patients with breast cancer – results of an international multicenter cohort study

Alexander Fabian[1*], Daniel Buergy[2*], Fabian Weykamp[3,4,5,6], Juliane Hörner-Rieber [3,5,6,7], Denise Bernhardt[8], Judit Boda-Heggemann[2], Montserrat Pazos[9], Nora Mehrhof[10], David Kaul[10,11], Alicia S. Bicu[2], Eugenia Vlaskou Badra[12], Susanne Rogers[13], Stefan Janssen[14,15], Hossein Hemmatazad[16], Katharina Hintelmann[17], Eleni Gkika[18,19], Tim Lange[20], Konstantinos Ferentinos[21], Heiko Karle[22], Thomas Brunner[23], Andrea Wittig[24], Marciana Nona-Duma[25,26], Oliver Blanck[1], David Krug [1,17]

Corresponding author: Alexander Fabian, MD (alexander.fabian@uksh.de)
Department of Radiation Oncology, University Hospital Schleswig-Holstein Campus Kiel, Kiel, Germany
Arnold-Heller-Str.3, 24105 Kiel, Tel. +49-431-500-26501; Fax +49-431-500-26548

Supplementary Figure 1 Flow chart. Abbreviation: RT, radiotherapy

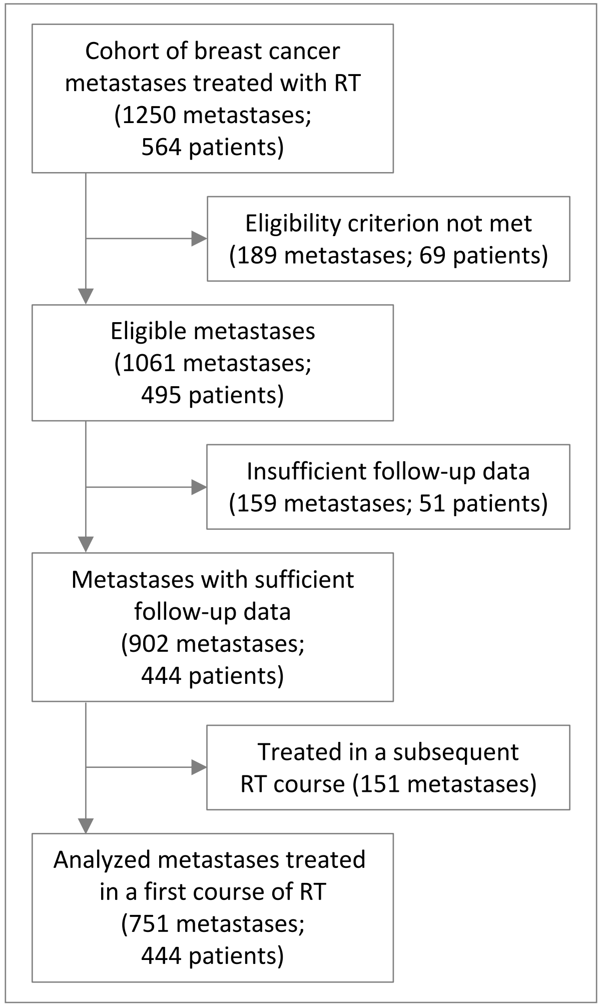

Supplementary Table 1 Characteristics of SRT for metastases (n=751) of breast cancer treated in a first course of metastasis-directed radiotherapy. Numbers given in brackets are absolute numbers. Abbreviations: BED, biologically equivalent dose, GTV, gross tumor volume; PTV, planning target volume; SD, standard deviation

		Intracranial (73%, 547/751)		Extracranial (27%, 204/751)[1]		
Site		Intact metastasis	Resection Cavity	Bone	Liver	Lung
Frequency		64% (482/751)	9% (65/751)	13% (96/751)	7% (51/751)	6% (48/751)
Fractionation regimen	Most common (n)	1 x 20 Gy (299)	7 x 5 Gy (33)	5 x 7 Gy (24)	5 x 12 Gy (8)<br>3 x 15 Gy (8)	5 x 12 Gy (11)
	2nd	1 x 18 Gy (47)	10 x 4 Gy (7)<br>5 x 6 Gy (7)	3 x 9 Gy (17)	5 x 9 Gy (6)	3 x 15 Gy (6)<br>8 x 7.5 Gy (6)
	3rd	6 x 5 Gy (30)	1 x 20.48 Gy (5)	5 x 6 Gy (10)<br>5 x 8 Gy (10)	1 x 24 Gy (5)<br>1 x 28 Gy (5)	12 x 5 Gy (4)
	Other	(106)	(13)	(35)	(19)	(21)
Prescribed dose (BED10)	mean ± SD, Gy	58.6 ± 7.2	53.2 ± 5.2	58.2 ± 8.4	100.1 ± 23.3	102.0 ± 22.5
Size of GTV	mean ± SD, cc	1.3 ± 2.0	13.9 ± 16.6[2]	13.0 ± 20.7	27.2 ± 49.5	6.8 ± 11.8
Size of PTV	mean ± SD, cc	2.9 ± 5.7	25.4 ± 25.9	44.9 ± 65.1	69.4 ± 63.4	26.7 ± 28.4

[1] Other extracranial sites comprise 1 % (9/751) of the dataset.

[2] Size of the resection cavity
